# Therapeutic Potential of Hydrazones as Anti-Inflammatory Agents

**DOI:** 10.1155/2014/761030

**Published:** 2014-03-04

**Authors:** Anu Kajal, Suman Bala, Neha Sharma, Sunil Kamboj, Vipin Saini

**Affiliations:** M. M. College of Pharmacy, Maharishi Markandeshwar University, Mullana, Ambala, Haryana 133207, India

## Abstract

Hydrazones are a special class of organic compounds in the Schiff base family. Hydrazones constitute a versatile compound of organic class having basic structure (R_1_R_2_C=NNR_3_R_4_). The active centers of hydrazone, that is, carbon and nitrogen, are mainly responsible for the physical and chemical properties of the hydrazones and, due to the reactivity toward electrophiles and nucleophiles, hydrazones are used for the synthesis of organic compound such as heterocyclic compounds with a variety of biological activities. Hydrazones and their derivatives are known to exhibit a wide range of interesting biological activities like antioxidant, anti-inflammatory, anticonvulsant, analgesic, antimicrobial, anticancer, antiprotozoal, antioxidant, antiparasitic, antiplatelet, cardioprotective, anthelmintic, antidiabetic, antitubercular, trypanocidal, anti-HIV, and so forth. The present review summarizes the efficiency of hydrazones as potent anti-inflammatory agents.

## 1. Introduction

Inflammation is a physiological reactionwhich involves cellular and biochemical responses, which is not only symptom for common diseases but also known to be an early phase for some serious diseases such as alzheimer's disease, cancer, heart vascular diseases [1] etc. Nonsteroidal anti-inflammatory drugs (NSAIDs) like ketoprofen, ibuprofen, aceclofenac, and so forth under current clinical usage for the treatment of inflammation, algesia and pyresis [2] are associated with major drawbacks of gastrointestinal disorders like dyspepsia, gastric ulcers, and so forth, due to the direct contact of free carboxylic group with the gastric mucosa [3, 4] and due to decrease in production of prostaglandins in tissue [5]. In order to overcome these drawbacks, there is an urgent need for design and synthesis of new chemical entities with excellent anti-inflammatory response and minimum side effects. Hydrazones are a class of organic compounds in the Schiff base family [6]. Hydrazones constitute a versatile compound of organic class having the basic structure R_1_R_2_C=NNR_3_R_4_ [7, 8]. Two nitrogen atoms of hydrazone are nucleophilic but the amino type nitrogen is more reactive, whereas the carbon atom possesses both characters, that is, nucleophilic and electrophilic. The active centers of hydrazine, that is, carbon and nitrogen, are mainly responsible for the physical and chemical properties of the hydrazones and, due to the reactivity toward electrophiles and nucleophiles, hydrazones are used for the synthesis of organic compound such as heterocyclic compounds [9, 10]. The general method for the synthesis of the hydrazones is the reaction of hydrazine with carbonyl compounds such as aldehydes or ketones in solvents like ethanol, methanol, butanol [11–13], and so forth. Hydrazones and their derivatives are known to exhibit interesting diverse biological activities like antioxidant [14], anti-inflammatory [15, 16], anticonvulsant [17, 18], analgesic [19, 20], antimicrobial [21–23], anticancer [24, 25], antiprotozoal [26], antiparasitic [27], cardioprotective [28], antidepressant [29], antitubercular [30, 31], anti-HIV [32], and trypanocidaletc. Hydrazones are also emerging as moiety of interest in medical biotechnology. Hydrazones are also used to couple with certain drugs and the bonds based on hydrazones are stable at the neutral pH [33]. The hydrazone Schiff bases of aroyl, acyl, and heteroaroyl compounds are known to have an additional donor site, that is, C=O, which make them more versatile and flexible. This versatility has leaded the hydrazones to emerge as good chelating agents that can form a variety of complexes with different transition metals [6]. Some hydrazones have been introduced by the researchers as potent drugs, such as nifuroxazide, an intestinal antiseptic [34, 35], dihydralazine as hypertensive, and gyromitrin, a toxin.

## 2. Anti-Inflammatory Activity

**Figure 1:**
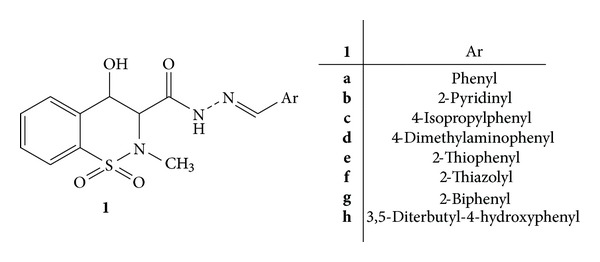


A series of benzothiazine N-acylhydrazones **1**(**a–h**) was designed by structural modification of piroxicam and synthesized and evaluated for the anti-inflammatory and antinociceptive activities (Figure 1). The pharmacological screening revealed that compounds **1**(**a–h**) have exhibited better activity than standard drug piroxicam. Compounds **1f **and **1g** were identified as new anti-inflammatory and antinociceptive agents which were capable of inhibiting cell recruitment by 70% and 80%, respectively, at dose of 100 *μ*mol/kg, p.o in zymosan- and carrageenan-induced peritonitis [36].

**Figure 2:**
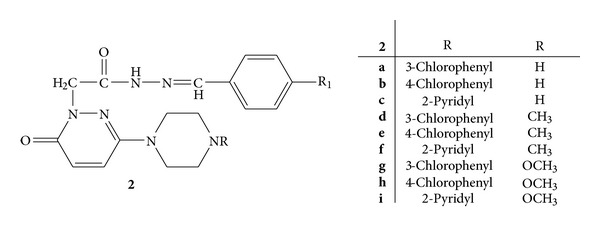


A novel series of 6-substituted-3(2H)-pyridazinone-2-acetyl-2(p-substituted/nonsubstituted benzal)hydrazine **2**(**a–i**) was synthesized and evaluated for their analgesic and anti-inflammatory activity (Figure 2). The activity was evaluated by using carrageenan-induced paw oedema assay and indomethacin was employed as standard for comparison of results. The results revealed that 6-substituted-3(2H)-pyridazinone-2-acetyl-2(nonsubstitutedbenzal)hydrazones, that is, **2a**, **2b**, and **2c**, were found to be most potent anti-inflammatory agents. Compound **2a**, 6-[4-(3-chlorophenyl)piperazine]-3(2H)-pyridazinone-2-acetyl-2(p-substituted/nonsubstituted benzal)hydrazine, was found to be slightly better than standard drug indomethacin [37].

**Figure 3:**
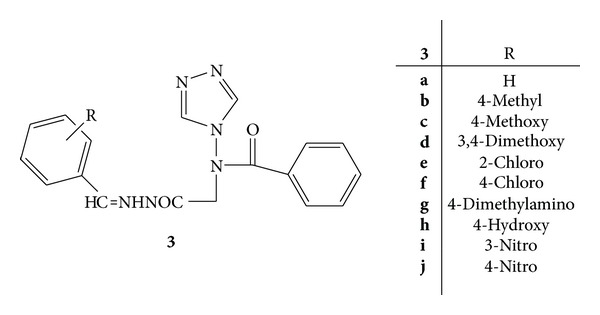


A novel series of N-(substituted benzylidene)-2-(N-(4H-1,2,4-triazole-4-yl) benzamido)acetohydrazide derivatives **3**(**a–j**) was synthesized and evaluated for the anti-inflammatory and antimicrobial activity (Figure 3). Indomethacin was used as standard drug and carrageenan-induced paw oedema method was employed for the anti-inflammatory screening of the test compounds. It was found that compounds **3f**, **3g**, and **3j **substituted with 4-chloro, 4-dimethylamino, and 4-nitro exhibited 66.7, 61.7, and 63.2% inhibition, respectively, at 50 mg/kg, oral dose, whereas standard drug indomethacin had shown 64.2% inhibition at 10 mg/kg, oral dose [38].

**Figure 4:**
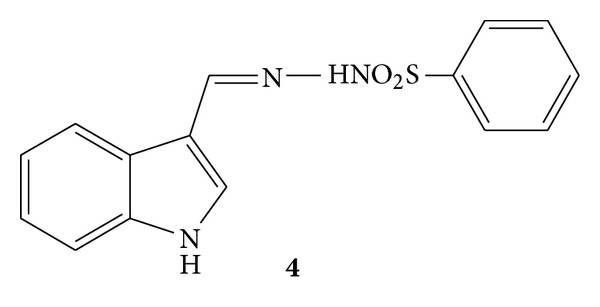


Synthesis of a novel series of some amidine and hydrazone derivatives **4** was reported (Figure 4). The synthesized compounds were further subjected to evaluation of anti-inflammatory and analgesic activities. Anti-inflammatory activity evaluation was carried out using carrageenan-induced rat paw oedema assay and compound **4a **was found to have good anti-inflammatory activity [39].

**Figure 5:**
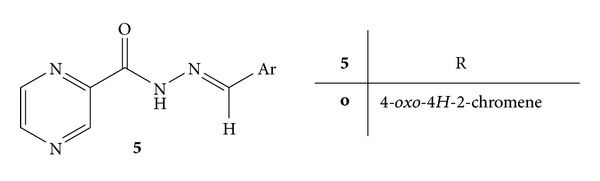


A series of nineteen pyrazine N-acylhydrazone (NAH) derivatives **5**(**a–s**) was (Figure 5) designed by molecular simplification of the prototype (LASSBio-1018), a nonselective cyclooxygenase inhibitor. Synthesis of the designed compounds was carried out and they were evaluated for their anti-inflammatory and analgesic activities in several animal models of pain and inflammation like writhing test, formalin test, hot plate test, zymosan-induced peritonitis, capsaicin-induced ear edema, and Freund's adjuvant-induced arthritis model. Thalidomide (TNF-*α* inhibitor), celecoxib (COX-2) inhibitor, and indomethacin (selective COX-1 inhibitor) were employed as standard drugs. Results enlightened that compound **5o** (2-*N*′-[(E)-(3,4,5-trimethoxyphenyl)methylidene]-2-pyrazinecarbohydrazide, LASSBio-1181, had better pharmacological activities and can be used as a lead compound for development of new analgesic and anti-inflammatory agents [40].

**Figure 6:**
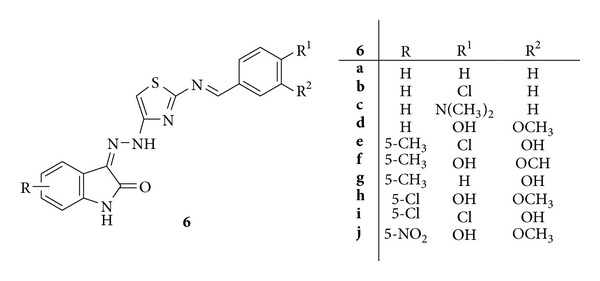


A novel series of isatin derivatives, that is, isatin-3-[N2-(2-benzalaminothiazol-4-yl)]hydrazones **6**(**a–j**), was synthesized and evaluated for anti-inflammatory, analgesic, and antipyretic activities (Figure 6) [41].

Carrageenan-induced rat paw oedema model was used for evaluation of anti-inflammatory activity and indomethacin was employed as standard drug. It was found that the compounds **6f, 6h**, and** 6j** substituted at fifith position with methyl, chloro, and nitro groups, respectively, exhibited significant anti-inflammatory activity [41].

**Figure 7:**
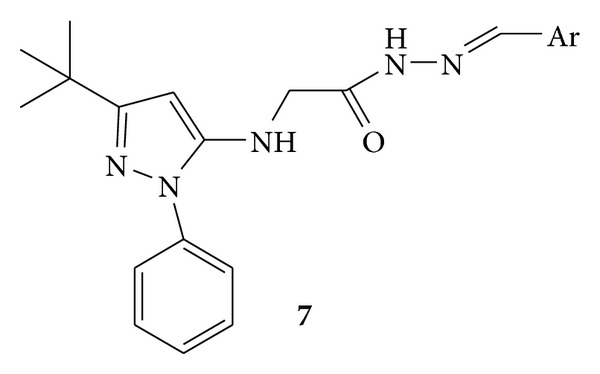


**Figure 8:**
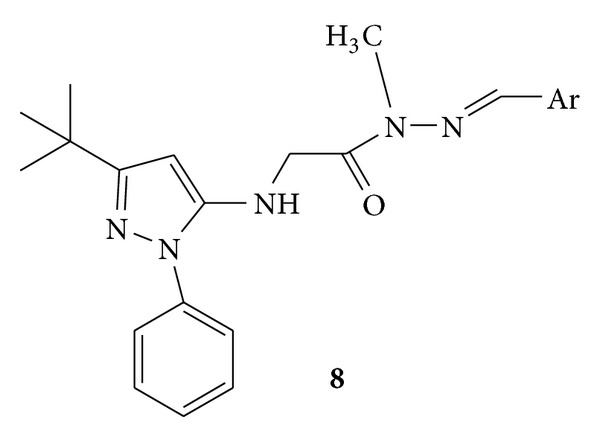


Synthesis of a novel series of orally active N-phenylpyrazolyl-N-glycinyl-hydrazone derivatives, **7a–g** (Figure 7) and **8 **(Figure 8), was carried out. Synthesized compounds were evaluated for their *in vivo* analgesic and anti-inflammatory activities and *in vitro* inhibition of TNF-*α* (tumor necrosis factor). Compounds **7a**, (E)-2-(3-tert-butyl-1-phenyl-1H-pyrazol-5-ylamino)-*N*′-((4-(2-morpholinoethoxy)naphthalen-1-yl) methylene)acetohydrazide, and **7f**, (E)-2-(3-tert-butyl-1-phenyl-1H-pyrazol-5-ylamino)-N′-(4-chlorobenzylidene)acetohydrazide, were known to possess anti-inflammatory activities when compared to the standard drug used, that is, SB-203580 [42].

**Figure 9:**
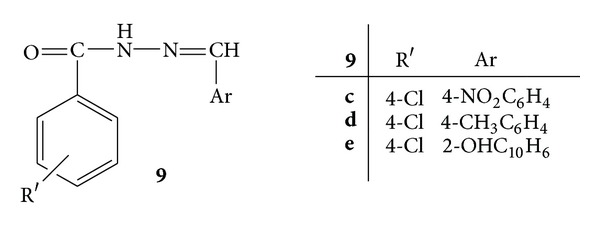


Synthesis of a novel series of substituted-*N*′-[(1E)-substituted phenylmethylidene]benzohydrazide analogs, **9**(**a–n**) (Figure 9) was reported and evaluated for their *in vitro* anti-inflammatory, antimicrobial and antioxidant activities. Albumin denaturation studies were carried out in order to evaluate the anti-inflammatory activity by employing diclofenac sodium as standard. Compounds **9c**,** 9d**, and** 9e** were reported to have good anti-inflammatory activity due to presence of 4-nitro, 4-methyl, and 2-hydroxy groups, respectively, whereas **9e** was found to be the most active anti-inflammatory agent [43].

**Figure 10:**
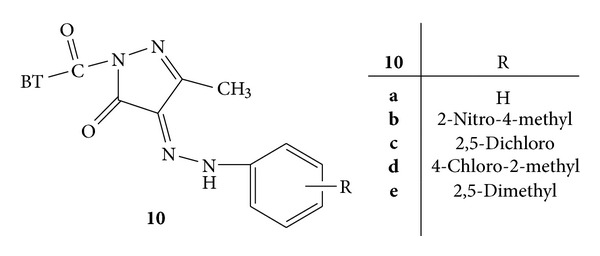


Synthesis and biological evaluation of some new benz[b]thiophene derivatives like thiadiazole, pyrazoline, oxadiazole and diaryl pyrazoles was carried out. N^1^-(3- chlorobenzo[b]thiophene-2-carbonyl)-3-methyl-4-9 substituted phenylhydrazono pyrazolin-5-one **10(a-e)** were subjected to anti-inflammatory and antimicrobial evaluation (Figure 10). Compound **10b** substituted with 2-nitro-4-methyl was found to have 50.25 % inhibition which is near about the standard drug diclofenac sodium i.e. 51.88% [44].

Synthesis of zinc complexes **11, **[Zn(LASSBio-466)H_2_O]_2_ of salicylaldehyde 2-chlorobenzoyl hydrazone (H_2_LASSBio-466), and **12**, [Zn(HLASSBio-1064)Cl]_2_ salicylaldehyde 4-chlorobenzoyl hydrazone (H_2_LASSBio-1064), was carried out. The complexes were further subjected to evaluation for peripheral and central nociception and acute inflammation in animal models. Both of the complexes exhibited anti-inflammatory activity. Complex **11 **has shown activity in both phases of formalin test like indomethacin and indicated its ability to inhibit nociception associated with the inflammatory response, whereas H_2_LASSBio-466 was active only in first phase of formalin test. H_2_LASSBio-1064 inhibited both phases but no improvement was indicated by the complex [45].

**Figure 11:**
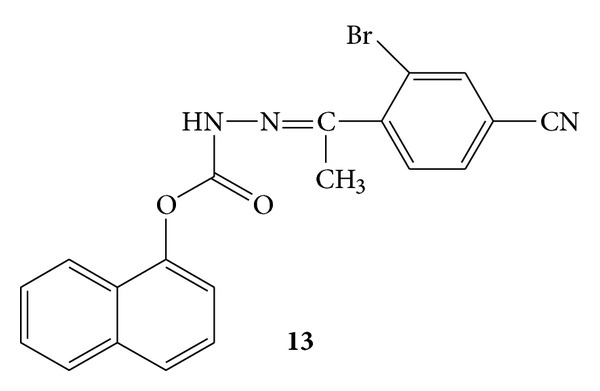


Synthesis of some new hydrazide derivatives of 2-napthoxy acetic acid [46], nicotinic acid [47, 48] and napthlene-1-acetic acid [49] has been reported. Out of these hydrazide derivatives most active antimicrobial compounds were selected and subjected to evaluation of the anti-inflammatory activity. Carrageenan induced rat paw oedema model was employed for the evaluation of theses active compounds using diclofenac sodium as standard drug. Results revealed that naphthalen-1-yloxy)-acetic acid [1-(2-bromo-4-cyano-phenyl)-ethylidene]-hydrazide **13 **(Figure 11) has shown percent inhibition of 20.90% at a dose of 50 mg/kg [50].

**Figure 12:**
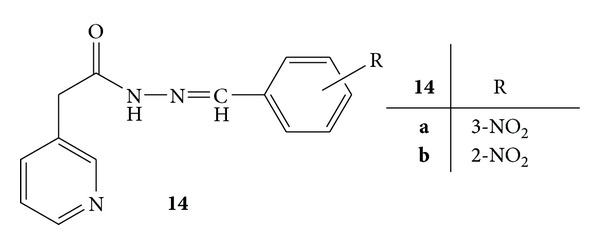


The nicotinic acid hydrazide derivatives, **14a** and **14b**, substituted with nitro group at *meta* and *ortho* positions, respectively, were found to be the most active anti-inflammatory agents (Figure 12). The percent inhibition of compounds **14a** and **14b** was found to be 37.29% and 35.73%, respectively, at the dose of 20 mg/kg and 34.17% and 25.12%, respectively, at the dose of 50 mg/kg, whereas percent inhibition of diclofenac sodium was found to be 38.85%. The conclusion drawn from the results was that the substitution of nitro group and halogens contributed to anti-inflammatory activity [50].

**Figure 13:**
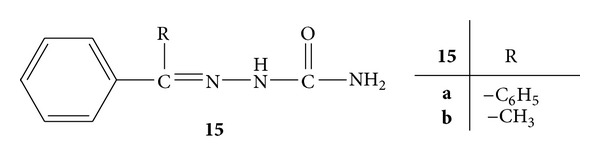


Synthesis of benzophenone semicarbazone (BSC) **15a **and acetophenone semicarbazone (ASC) **15b **was carried out (Figure 13). The anti-inflammatory activity was determined on Swiss albino mice by using carrageenan-induced mice paw oedema model. Both of the compounds were screened at two different doses, that is, 25 mg/kg and 50 mg/kg (p.o.). Compound **15a **showed 36.6% and 46.6% of inhibition at 25 mg/kg and 50 mg/kg, respectively, whereas compound **15b **showed 34.6% and 41.5% inhibition. Diclofenac sodium was used as standard drug which showed 70.29% inhibition at 10 mg/kg (p.o.). From the above observations, it was concluded that both of the test compounds possessed anti-inflammatory activity [51].

**Figure 14:**
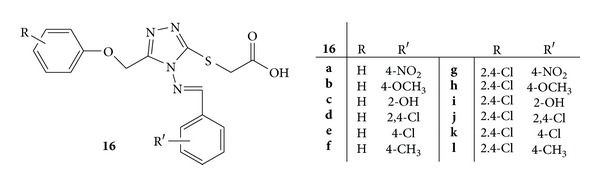


A novel series of 2-[4-(substituted benzylideneamino)-5-(substituted phenoxymethyl)-4H-1,2,4-triazol-3-yl thio]acetic acid, **16**(**a–l**), was synthesized (Figure 14). All the newly synthesized compounds were evaluated for *in vivo *anti-inflammatory and analgesic activities. Among the series **16d**, **16e**, **16j**, and **16k** showed significant activity with 63.4%, 62.0%, 64.1%, and 62.5% edema inhibition, respectively, as compared to standard drug diclofenac 67.0% after the third hour. Compounds **16g**, **16h**, **16i**, and **16l** showed good anti-inflammatory activity, whereas compounds **16a–c** and **16f** were found to be the least active. The results enlightened the effect of electron withdrawing moiety, that is, chloro group on the anti-inflammatory activity [52].

**Figure 15:**
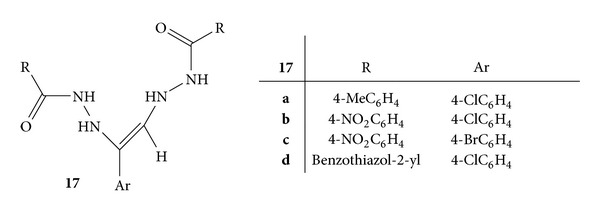


A series of bis-hydrazone derivatives, that is, (Z)-N′,N′′-(1-(4-substituted phenyl)ethene-1,2-diyl)bis(4-substituted benzhydrazide), **17**(**a–d**), was synthesized by reaction of 2-chloro-1-(4-chloro phenyl)ethanone or 2-bromo-1-(4-bromophenyl)ethanone with acid hydrazides (Figure 15). All the synthesized compounds were evaluated for the anti-inflammatory, analgesic, and ulcerogenic activities. Formalin-induced rat paw oedema model was selected and ketoprofen was employed as standard drug. All compounds were found to exhibit good anti-inflammatory activity with percent inhibition of 68.4% and 61.4% of compounds **17b** and **17c**, respectively, after three hours [53].

**Figure 16:**
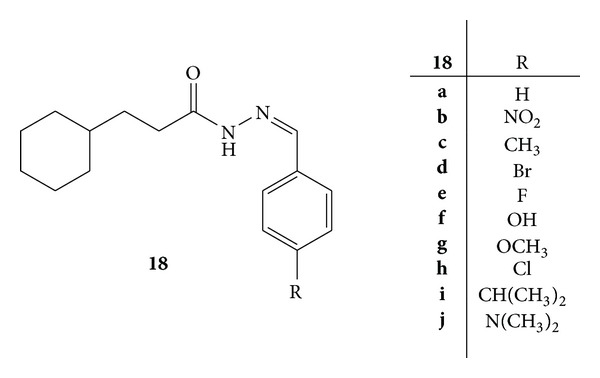


Synthesis of a series of hydrazone derivatives, that is, N-(substituted benzylidene)-3-cyclohexylpropionic acid hydrazide, **18(a–j)**, was carried out (Figure 16). The synthesized compounds were evaluated for anti-inflammatory activity and cytotoxicity. Inducible nitric oxide synthase (iNOS) and NF-*κ*B were selected for determination of anti-inflammatory activity. Inhibition of iNOS activity was also observed in LPS-induced RAW 264.7 cells. Compounds **18c**, **18e**, and **18i** were more active than the other synthesized compounds which proved that there is a positive correlation between the inhibitions of iNOS activity and functional groups, that is, methyl, fluoro, and isopropyl, on phenyl ring [54].

**Figure 17:**
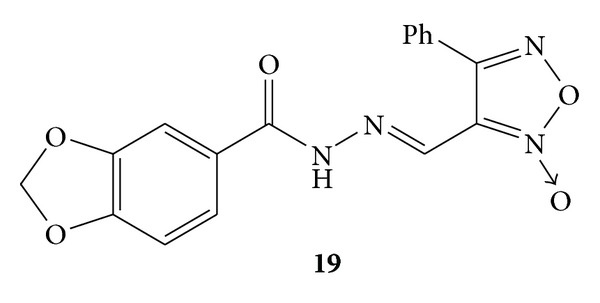


Inhibition of NF- *κ*B mediated transcription was seen in human chondrosarcoma (SW1353) cells and compounds **18a**, **18c**, and **18h** have shown inhibition with IC_50_ values of 6.9, 7.7, and 6.4 *μ*g/mL, respectively (Figure 17). This observation correlated that methyl and chloro groups have a considerable influence on the inhibition of NF-*κ*B mediated transcription. The other compounds **18d**, **18e**, **18g**, and **18i** inhibited the NF-*κ*B activity to a lesser extent with IC_50_ values in the range of 10–14 *μ*g/mL. Compounds **18b**, **18d**, and **18j** were found to be comparatively less active than other compounds. From the results, it was concluded that substitutions at *para* position of phenyl ring had significant influence on anti-inflammatory activity [54].

**Figure 18:**
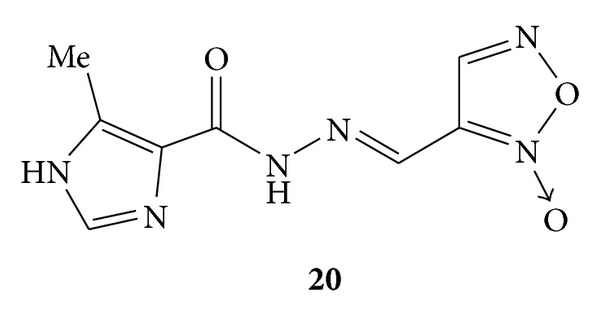


**Figure 19:**
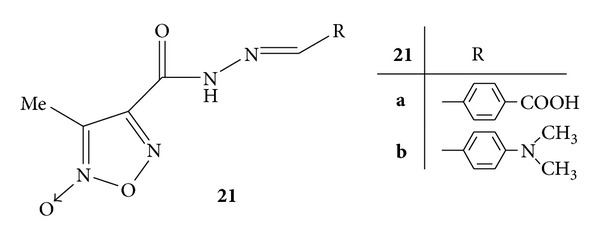


By application of molecular hybridization approach design, and synthesis of thirty two furoxanyl N-acylhydrazones(furoxanyl-NAH) was carried out. Synthesized compounds were evaluated for their *in vitro* as well as *in-vivo* analgesic and anti-inflammatory activities. The *in vitro* anti-inflammatory activity was evaluated by decrease in NF-*κ*B activation and interleukin-8 inhibition by using a human pathway-specific reporter cell system (HT-29-NF-*κ*B-hrGFP) whereas carrageenan induced paw oedema was used for *in vivo* evaluation of anti-inflammatory activity. Furoxanyl-NAH **19** (Figure 17)and benzofuroxanyl-derivative **20 **(Figure 18) were reported to have orally anti-inflammatory and analgesic activities without interleukin-8 inhibition. Furoxanyl-NAH derivative **21a **(Figure 19)was emerged as a structural lead to develop new lipoxygenase (LOX) inhibitors. The active derivatives **19**, **21a** and **21b** were found to be less mutagenic and were proposed as candidates for the further clinical studies [55].

Synthesis of a series of novel acyl-hydrazones bearing 2-aryl-thiazole moiety was carried out. The synthesized compounds were screened for *invivo* anti-inflammatory activity by evaluating three parameters, that is, nitric oxide synthesis, phagocytes activity, and acute phase bone marrow response, in acute experimental inflammation [56].

**Figure 20:**
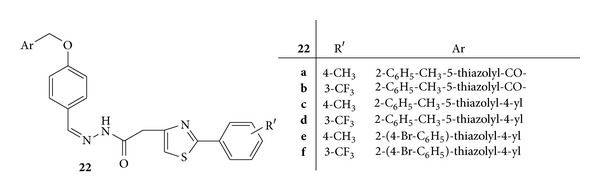


Compounds **22c**,** 22e**,** 22f**,** 24b**, and** 26b** were found to have a good inhibitory effect on the acute phase marrow response by reducing the absolute leukocytes count due to the lower neutrophils percentage. Compound **22c** (Figure 20) with 2-phenyl-thiazole and [2-(4-methylphenyl)-4-methylen]-thiazole hydrazine moieties was proved to be a more potent inhibitor of the acute phase bone marrow response than meloxicam, the anti-inflammatory standard drug [56].

**Figure 21:**
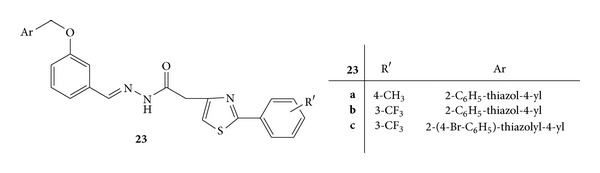


**Figure 22:**
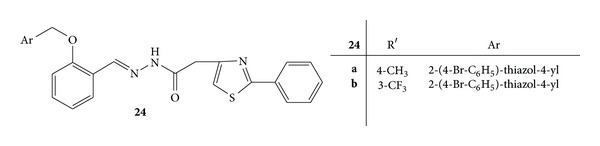


**Figure 23:**
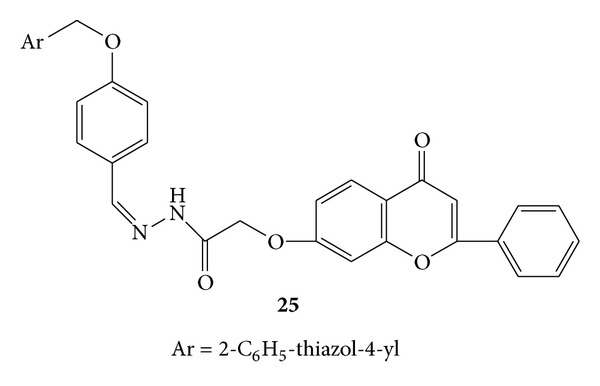


**Figure 24:**
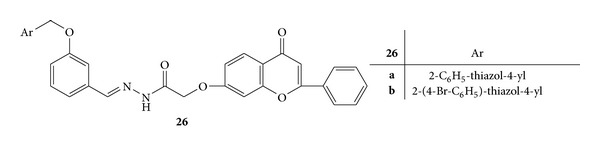


Phagocytic activity was assessed by calculating phagocytic index (PI) and the phagocytic activity (PA). All the newly synthesized compounds reduced PI significantly and compounds **22a **(Figure 20),** 22b **(Figure 20), **22d **(Figure 20),** 23b **(Figure 21),** 24b **(Figure 22),** 25 **(Figure 23)** and 26b **(Figure 24)were observed as more potent inhibitors than meloxicam. PA was significantly reduced by the compounds **22a**,** 22d**,** 23b**,** 24b**,** 23c**,** 25 **and** 26b **from which** 22a**,** 22d**,** 20 **and** 26b** were found to be more potent inhibitors than meloxicam [56].

Synthesis of NO increases significantly in acute inflammation due to the expression of iNOS. The NO synthesis was significantly reduced by **22a**,** 22b**,** 23c**,** 26a**, and** 26b **and they all, except for** 26a**, displayed a stronger inhibitory activity than meloxicam [56].

**Figure 25:**
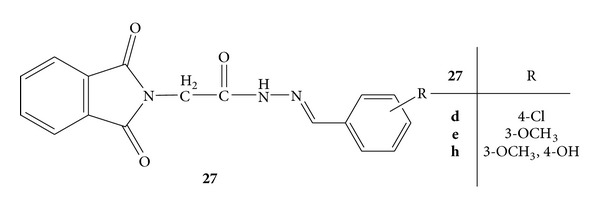


Synthesis of a novel series of phthalic anhydride based substituted benzylidene-hydrazide derivatives, **27a–i**, was carried out. All the synthesized derivatives were screened for *in vivo *anti-inflammatory and analgesic activities by carrageenan-induced rat paw oedema and tail immersion methods, respectively, using diclofenac sodium as standard drug. The results revealed that derivatives **27d**, **27e**, and **27h** (Figure 25) have shown potent anti-inflammatory activity with percentage inhibition of 58.6%, 61.4%, and 64.0%, respectively, which is comparable with standard drug diclofenac sodium, that is, 68.0%. The reaction time of derivatives **27d **and** 27h **that was found to be 8.91 ± 0.21 and 9.09 ± 0.03, respectively, after 90 minutes which is comparable with reaction time of diclofenac sodium (10.93 ± 0.01) after 90 minutes has shown analgesic potency of these derivatives [57].

## 3. Conclusion

Hydrazone derivatives are well known to have various important pharmacological activities and are used for synthesis of a wide variety of medicinally active compounds. This review paper summarizes the anti-inflammatory potential of hydrazone derivatives and the effect of substitutions of different groups on the anti-inflammatory activity. This summarized study is an attempt to bring about the anti-inflammatory activity for awaking the safe use of this important chemical moiety with minimal or no ulcerogenic effects in future.

## References

[1] Ingale N, Maddi V, Palkar M (2012). Synthesis and evaluation of anti-inflammatory and analgesic activity of 3-[(5-substituted-1,3,4-oxadiazol-2-yl-thio)acetyl]-2*H*-chromen-2-ones. *Medicinal Chemistry Research*.

[2] Rigas B (2007). The use of nitric oxide-donating nonsteroidal anti-inflammatory drugs in the chemoprevention of colorectal neoplasia. *Current Opinion in Gastroenterology*.

[3] Venerito M, Wex T, Malfertheiner P (2010). Nonsteroidal anti-inflammatory drug-induced gastroduodenal bleeding: risk factors and prevention strategies. *Pharmaceuticals*.

[4] Lanas A, García-Rodríguez LA, Arroyo MT (2007). Effect of antisecretory drugs and nitrates on the risk of ulcer bleeding associated with nonsteroidal anti-inflammatory drugs, antiplatelet agents, and anticoagulants. *The American Journal of Gastroenterology*.

[5] Prakash S, Gupta BN, Moorthy NSH (2007). Synthesis and physicochemical characterization ofmutual prodrug of indomethacin. *Trends in Applied Science Research*.

[6] Cornelissen JP, Van Diemen JH, Groeneveld LR, Haasnoot JG, Spek AL, Reedijk J (1992). Synthesis and properties of isostructural transition-metal (copper, nickel, cobalt, and iron) compounds with 7,7′,8,8′-tetracyanoquinodimethanide(1-) in an unusual monodentate coordination mode. Crystal structure of bis(3,5-bis(pyridin-2-yl)-4-amino-1,2,4-triazole)bis(7,7′,8,8′- tetracyanoquinodimethanido)copper(II). *Inorganic Chemistry*.

[7] Smith MB, Jerry M (2007). *March’s Advanced Organic Chemistry: Reactions, Mechanisms, and Structure*.

[8] Lazny R, Nodzewska A (2010). *N*,*N*-dialkylhydrazones in organic synthesis. From simple *N*,*N*-dimethylhydrazones to supported chiral auxiliaries. *Chemical Reviews*.

[9] Belskaya SNP, Dehaen W, Bakuleva VA (2010). Synthesis and properties of hydrazones bearing amide, thioamide and amidine functions. *Arkivoc*.

[10] Brehme R, Enders D, Fernandez R, Lassaletta JM (2007). Aldehyde *N*,*N*-dialkylhydrazones as neutral acyl anion equivalents: umpolung of the imine reactivity. *European Journal of Organic Chemistry*.

[11] Berdinskii IS (1979). The chemistry of hydrazones. *Chemistry of Heterocyclic Compounds*.

[12] Smith PAS (1983). Hydrazones. *Derivatives of Hydrazine and Other Hydronitrogens Having N-N Bonds*.

[13] Polshettiwar V, Varma RS (2007). Polystyrene sulfonic acid catalyzed greener synthesis of hydrazones in aqueous medium using microwaves. *Tetrahedron Letters*.

[14] Belkheiri N, Bouguerne B, Bedos-Belval F (2010). Synthesis and antioxidant activity evaluation of a syringic hydrazones family. *European Journal of Medicinal Chemistry*.

[15] Radwan MAA, Ragab EA, Sabry NM, El-Shenawy SM (2007). Synthesis and biological evaluation of new 3-substituted indole derivatives as potential anti-inflammatory and analgesic agents. *Bioorganic & Medicinal Chemistry*.

[16] Almasirad A, Tajik M, Bakhtiari D (2005). Synthesis and analgesic activity of N-arylhydrazone derivatives of mefenamic acid. *Journal of Pharmacy & Pharmaceutical Sciences*.

[17] Sridhar SK, Pandeya SN, Stables JP, Ramesh A (2002). Anticonvulsant activity of hydrazones, Schiff and Mannich bases of isatin derivatives. *European Journal of Pharmaceutical Sciences*.

[18] Kaushik D, Khan SA, Chawla G, Kumar S (2010). *N*’-[(5-chloro-3-methyl-1-phenyl-1*H*-pyrazol-4-yl)methylene] 2/4-substituted hydrazides: synthesis and anticonvulsant activity. *European Journal of Medicinal Chemistry*.

[19] Duarte CD, Barreiro EJ, Fraga CAM (2007). Privileged structures: a useful concept for the rational design of new lead drug candidates. *Mini-Reviews in Medicinal Chemistry*.

[20] Salgin-Gökşen U, Gökhan-Kelekçi N, Göktaş Ö (2007). 1-Acylthiosemicarbazides, 1,2,4-triazole-5(4*H*)-thiones, 1,3,4-thiadiazoles and hydrazones containing 5-methyl-2-benzoxazolinones: synthesis, analgesic-anti-inflammatory and antimicrobial activities. *Bioorganic & Medicinal Chemistry*.

[21] Deeb A, El-Mariah F, Hosny M (2004). Pyridazine derivatives and related compounds—part 13: synthesis and antimicrobial activity of some pyridazino[3′,4′:3,4]pyrazolo[5,1-*c*]- 1,2,4-triazines. *Bioorganic & Medicinal Chemistry Letters*.

[22] Rasras AJM, Al-Tel TH, Al-Aboudi AF, Al-Qawasmeh RA (2010). Synthesis and antimicrobial activity of cholic acid hydrazone analogues. *European Journal of Medicinal Chemistry*.

[23] Özkay Y, Tunali Y, Karaca H, Işikdağ I (2010). Antimicrobial activity and a SAR study of some novel benzimidazole derivatives bearing hydrazone moiety. *European Journal of Medicinal Chemistry*.

[24] Kumar D, Kumar NM, Ghosh S, Shah K (2012). Novel bis(indolyl)hydrazide-hydrazones as potent cytotoxic agents. *Bioorganic & Medicinal Chemistry Letters*.

[25] Effenberger K, Breyer S, Schobert R (2010). Modulation of doxorubicin activity in cancer cells by conjugation with fatty acyl and terpenyl hydrazones. *European Journal of Medicinal Chemistry*.

[26] Caputto ME, Fabian LE, Benítez D (2011). Thiosemicarbazones derived from 1-indanones as new anti-*Trypanosoma* cruzi agents. *Bioorganic & Medicinal Chemistry*.

[27] Aslam MAS, Mahmood S-U, Shahid M, Saeed A, Iqbal J (2011). Synthesis, biological assay *in vitro* and molecular docking studies of new Schiff base derivatives as potential urease inhibitors. *European Journal of Medicinal Chemistry*.

[28] El-Sabbagh OI, Shabaan MA, Kadry HH, Al-Din ES (2010). New octahydroquinazoline derivatives: synthesis and hypotensive activity. *European Journal of Medicinal Chemistry*.

[29] Catto M, Aliano R, Carotti A (2010). Design, synthesis and biological evaluation of indane-2-arylhydrazinylmethylene-1,3-diones and indol-2-aryldiazenylmethylene-3-ones as *β*-amyloid aggregation inhibitors. *European Journal of Medicinal Chemistry*.

[30] Jordão AK, Sathler PC, Ferreira VF (2011). Synthesis, antitubercular activity, and SAR study of N-substituted- phenylamino-5-methyl-1*H*-1,2,3-triazole-4-carbohydrazides. *Bioorganic & Medicinal Chemistry*.

[31] Mahajan A, Kremer L, Louw S, Guéradel Y, Chibale K, Biot C (2011). Synthesis and *in vitro *antitubercular activity of ferrocene-based hydrazones. *Bioorganic & Medicinal Chemistry Letters*.

[32] Jin CAY, Tan Z, He M (2010). SAR and molecular mechanism study of novel acylhydrazone compounds targeting HIV-1 CA. *Bioorganic & Medicinal Chemistry*.

[33] Wu AM, Senter PD (2005). Arming antibodies: prospects and challenges for immunoconjugates. *Nature Biotechnology*.

[34] Enders D, Eichenauer H (1977). Enantioselective alkylation of aldehydes via metalated chiral hydrazones. *Tetrahedron Letters*.

[35] Livermore DM (2000). Antibiotic resistance in *staphylococci*. *International Journal of Antimicrobial Agents*.

[36] de Miranda AS, Júnior  WB, da Silva YKC (2012). Design, synthesis, antinociceptive and anti-inflammatory activities of novel piroxicam analogues. *Molecules*.

[37] Mehtap G, Semra U, Esra K (2009). Synthesis and analgesic and anti-inflammatory activities 6-substituted-3(2*H*)-pyridazinone-2-acetyl-2-(*p*-substituted/nonsubstituted benzal)hydrazone derivatives. *European Journal of Medicinal Chemistry*.

[38] Wahi AK, Singh AK, Singh A (2011). Design and synthesis of novel schiff’s bases having N-(4H-1, 2,4-triazole-4-yl)benzamido moiety as antimicrobial and anti-inflammatory agents. *Der Pharma Chemica*.

[39] Sondhi SM, Dinodia M, Kumar A (2006). Synthesis, anti-inflammatory and analgesic activity evaluation of some amidine and hydrazone derivatives. *Bioorganic & Medicinal Chemistry*.

[40] da Silva YKC, Augusto CV, Barbosa MLDC (2010). Synthesis and pharmacological evaluation of pyrazine *N*-acylhydrazone derivatives designed as novel analgesic and anti-inflammatory drug candidates. *Bioorganic & Medicinal Chemistry*.

[41] Venkateshwarlu E, Rao JV, Umasankar K, Dheeraj G (2012). Study of anti-inflammatory, analgesic and antipyretic activity of novel isatin derivatives. *Asian Journal of Pharmaceutical and Clinical Research*.

[42] Lacerda RB, da Silva LL, de Lima CKF (2012). Discovery of novel orally active anti-inflammatory *N*-phenylpyrazolyl-*N*-glycinyl-hydrazone derivatives that inhibit TNF-α production. *PLoS ONE*.

[43] Bala S, Uppal G, Kamboj S, Saini V, Prasad DN (2013). Design, characterization, computational studies, and pharmacological evaluation of substituted-*N*′-[(1E) substitutedphenylmethylidene]benzohydrazide analogs. *Medicinal Chemistry Research*.

[44] Isloor AM, Kalluraya B, Pai KS (2010). Synthesis, characterization and biological activities of some new benzo[b]thiophene derivatives. *European Journal of Medicinal Chemistry*.

[45] Júnior WB, Alexandre-Moreira MS, Alves MA (2011). Analgesic and anti-inflammatory activities of salicylaldehyde 2-chlorobenzoyl hydrazone (H2LASSBio-466), salicylaldehyde 4-chlorobenzoyl hydrazone (H2LASSBio-1064) and their zinc(II) complexes. *Molecules*.

[46] Narang R, Narasimhan B, Sharma S (2013). Substituted naphthalen-1-*γ*l-acetic acid hydrazides: synthesis, antimicrobial evaluation and QSAR analysis. *Medicinal Chemistry Research*.

[47] Narang R, Narasimhan B, Sharma S (2012). Synthesis, antimycobacterial, antiviral, antimicrobial activities, and QSAR studies of nicotinic acid benzylidene hydrazide derivatives. *Medicinal Chemistry Research*.

[48] Narang R, Narasimhan B, Sharma S (2011). Nicotinic acid benzylidene/phenyl-ethylidene hydrazides: synthesis, antimicrobial evaluation and QSAR studies. *Letters in Drug Design & Discovery*.

[49] Narang R, Narasimhan B, Sharma S (2012). (Naphthalen-1-yloxy)-acetic acid benzylidene/(1-phenyl-ethylidene)-hydrazide derivatives: synthesis, antimicrobial evaluation, and QSAR studies. *Medicinal Chemistry Research*.

[50] Narang R, Sharma S, Narasimhan B (2012). Evaluation of anti-inflammatory activity of acid Hydrazide derivatives. *Hygeia*.

[51] Ali SMM, Jesmin M, Azad MAK, Islam MK, Zahan R (2012). Anti-inflammatory and analgesic activities of acetophenonesemicarbazone and benzophenonesemicarbazone. *Asian Pacific Journal of Tropical Biomedicine*.

[52] Hunashal RD, Ronad PM, Maddi VS, Satyanarayana D, Kamadod MA (2011). Synthesis, anti-inflammatory and analgesic activity of 2-[4-(substituted benzylideneamino)-5-(substituted phenoxymethyl)-4*H*-1,2,4-triazol-3-yl thio] acetic acid derivatives. *Arabian Journal of Chemistry*.

[53] Hamdy NA, Abdel-Aziz HA, Kamel GM, Fakhr IMI (2013). Convenient synthesis, anti-inflammatory, analgesicand ulcerogenicactivites of some new bis-hydrazonesand pyrazole derivatives. *Acta Poloniae Pharmaceutica*.

[54] Kaplancikli ZA, Altintop MD, Özdemir A, Turan-Zitouni G, Khan SI, Tabanca N (2012). Synthesis and biological evaluation of some hydrazone derivatives as anti-inflammatory agents. *Letters in Drug Design & Discovery*.

[55] Hernández P, Cabrera M, Lavaggi ML (2012). Discovery of new orally effective analgesic and anti-inflammatory hybrid furoxanyl *N*-acylhydrazone derivatives. *Bioorganic & Medicinal Chemistry*.

[56] Moldovan CM, Oniga O, Pârvu A (2011). Synthesis and anti-inflammatory evaluation of some new acyl-hydrazones bearing 2-aryl-thiazole. *European Journal of Medicinal Chemistry*.

[57] Kajal A, Bala S, Kamboj S, Saini V (2013). Synthesis, characterization, and computational studies on phthalicanhydride-based benzylidene-hydrazide derivatives as novel, potential anti-inflammatory agents. *Medicinal Chemistry Research*.

